# In-Water Antibiotic Dosing Practices on Pig Farms

**DOI:** 10.3390/antibiotics10020169

**Published:** 2021-02-08

**Authors:** Stephen Little, Andrew Woodward, Glenn Browning, Helen Billman-Jacobe

**Affiliations:** 1 Asia Pacific Centre for Animal Health, Melbourne Veterinary School, Faculty of Veterinary and Agricultural Sciences, National Centre for Antimicrobial Stewardship, University of Melbourne, Parkville, VIC 3010, Australia; glenfb@unimelb.edu.au (G.B.); hbj@unimelb.edu.au (H.B.-J.); 2 Melbourne Veterinary School, Faculty of Veterinary and Agricultural Sciences, University of Melbourne, Parkville, VIC 3010, Australia; andrew.woodward@unimelb.edu.au

**Keywords:** swine, drinking water, antibiotic, systemic exposure, metaphylaxis, treatment, dosing pump, dosing regimen, antibiotic resistance

## Abstract

Pigs reared on many farms are mass-medicated for short periods with antibiotics through their drinking water to control bacterial pathogen loads and, if a disease outbreak occurs, to treat pigs until clinical signs are eliminated. Farm managers are responsible for conducting in-water antibiotic dosing events, but little is known about their dosing practices. We surveyed managers of 25 medium to large single-site and multi-site pig farming enterprises across eastern and southern Australia, using a mixed methods approach (online questionnaire followed by a one-on-one semi-structured interview). We found wide variation in the antibiotics administered, the choice and use of dosing equipment, the methods for performing dosing calculations and preparing antibiotic stock solutions, the commencement time and duration of each daily dosing event, and the frequency of administration of metaphylaxis. Farm managers lacked data on pigs’ daily water usage patterns and wastage and the understanding of pharmacology and population pharmacometrics necessary to optimize in-water dosing calculations and regimens and control major sources of between-animal variability in systemic exposure of pigs to antibiotics. There is considerable scope to increase the effectiveness of in-water dosing and reduce antibiotic use (and cost) on pig farms by providing farm managers with measurement systems, technical guidelines, and training programs.

## 1. Introduction

On many commercial pig farms, growing pigs are mass-medicated for short periods through their drinking water to manage herd health, productivity, and welfare [1,2,3]. Drinking water additives may include antibiotics, vaccines, parasiticides, organic acids, electrolytes, minerals, vitamins, amino acids, sweeteners, direct-fed microbials, essential oils, and potential new therapeutic products such as bacteriophages [2]. In-water dosing is well suited for two antibiotic use patterns in pigs; metaphylaxis and treatment. Metaphylaxis is ‘the mass treatment of animal populations currently experiencing any level of disease before the onset of blatant disease’. Treatment is the ‘administration of an antibiotic to an animal, or group of animals, which exhibits frank clinical disease’ [4]. Metaphylactic, in-water antibiotic dosing of pigs is conducted strategically when the target bacterial pathogen load is low. A short dosing period, administered once or at regular intervals, is intended to achieve a microbiological and clinical cure [5]. In-water antibiotic dosing of pigs to treat a disease outbreak is conducted for a short period until clinical signs disappear. A dosing event should result in the majority of pigs in a group attaining the level of systemic exposure to the antibiotic required to successfully eliminate or substantially reduce the quantity of the target pathogen and achieve high clinical efficacy, while minimizing selection for and propagation of resistant pathogens [6].

Antibiotic stewardship programs across food animal production sectors, including the pig industry, have been implemented in recent years in response to the increasing levels of antibiotic resistance found in human and veterinary medicine [7]. Strategies for antibiotic stewardship in pig production have been assisted by an increasing understanding of the antibiotic use patterns and prescribing behaviours of veterinarians and development of prescribing guidelines [8,9,10,11]. In Australia, antibiotics are not approved for use as growth promoters in pigs. On Australian pig farms, veterinarians must prescribe antibiotics to be administered and specify doses based on pig bodyweight. However, farm managers are usually responsible for conducting in-water antibiotic dosing events, and this involves making many decisions that may influence the actual dose administered and the subsequent systemic exposure of pigs in the group to the antibiotic. Despite the pivotal role of farm managers in the mass-medication of pigs with antibiotics through their drinking water, little is known about their choice and use of dosing equipment, the methods they use for making dosing calculations and preparing antibiotic stock solutions, the dosing regimens they use, the frequency of administration of metaphylaxis, and their views on successful in-water dosing.

We surveyed the managers of medium to large single-site and multi-site pig farming enterprises across eastern and southern Australia. A response rate of over 90% was achieved by directly contacting participants with the support of the owners and managers of the enterprises. We used a mixed methods approach, collecting and analysing both quantitative and qualitative data within the same study, as used by other researchers investigating antibiotic use [10,12]. A comprehensive online questionnaire was followed by a one-on-one, semi-structured interview with each farm manager. Our focus was on farm managers’ in-water antibiotic dosing practices. The pigs’ health status and performance, and the efficacy of the medication programmes were not assessed.

We discovered that in-water dosing practices were highly variable across the farms studied and that measurements were not being taken and used to assess the effectiveness of dosing events, optimize in-water dosing calculations and regimens, and control major sources of between-animal variability in systemic exposure of pigs to antibiotics.

## 2. Results

Twenty-five pig farm managers agreed to participate in the study. The participants’ farms were located in South Australia, Victoria, New South Wales, and Queensland. At the time of the study, these farms accommodated 459,167 weaner and grower/finisher pigs. This represents approximately 21% of all growing pigs accommodated in Australia at any one time [13]. The demographics of the sample population (participants and their farms) are presented in Appendix A, Table A1 and Table A2.

### 2.1. Dosing Equipment Used

The majority of farm managers surveyed used proprietary proportional dosing pumps to water medicate growing pigs, as shown in Table 1. The water-powered or electric-powered pumps inject a concentrated stock solution of antibiotic into the pig building’s water distribution system at a specified volumetric ratio e.g., one part stock solution to 100 parts water (commonly abbreviated to ‘1:100’). Managers reported that their choice of dosing equipment was guided by on-farm factors including the layout of drinking water distribution pipelines to buildings, the water pressure, building access to a mains power supply, the antibiotic products being used, the number of pigs to be dosed in each building, and past experiences with different brands and models of dosing pumps.

Many farm managers using dosing pumps previously used header tanks but had ceased using them because of concerns about (1) a lack of control and accuracy of dosing, (2) risks to workers’ health and safety associated with climbing ladders up to tanks, (3) the excessive time required to manage tanks, especially in large sheds, and (4) risks of staff members accidently depriving pigs of water after dosing by forgetting to re-open each tank’s inlet valve when the medicated solution was depleted.

Of the four farm managers using header tanks, three left each tank’s inlet valve open during dosing to allow water to flow into the top of tank as the medicated solution was drawn out the bottom of the tank. One manager was in the process of changing over to electric-powered dosing pumps. The other three managers had reduced the need for staff to climb ladders up to tanks by installing a canister system at ground level to transport antibiotic up a pipe into each header tank, but intended to change to dosing pumps in the near future.

*We clean (header tanks) in between batches. There’s always sediment in the bottom of the tank and material left over from the medication process. We know that it’s not an ideal system.*

Farm managers used proportional dosing pumps in different ways. Some installed them permanently in water lines to service one or more pig buildings. Others chose to re-locate them from building to building as required. On six managers’ farms, including two farms where dosing pumps were used to service multiple buildings, the antibiotic had to travel over 100 m from the dosing pump to some buildings. Two managers had opted to water medicate all weaner and grower/finisher buildings on the farm using a dedicated pipe system for medicated water from one or two dosing pumps located at a central point. On these two farms, the antibiotic had to travel a distance of between 70 and 135 m from the dosing pump to the most distant buildings. Farm managers were aware that locating a dosing pump a substantial distance from the pig building it served increased the antibiotic’s transit time from the pump to drinkers. However, they had not considered the potential implications of this, including the increased likelihood of degradation of the antibiotic.

*That’s the trade off when you’ve got one doser servicing six sheds rather than having the doser at each of the sheds.*

Across the farms, three brands of water-powered dosing pump were used: Dosatron (Dosatron International, Tresses, France); Gator-XL (Diemold International, Inc., Fort Myers, FL, USA); and Chemilizer (Hydro Systems, Cincinnati, OH, USA). All farms using electric-powered dosing pumps used the same brand, Select (Dosing Solutions Ltd., Clavering, Saffron Walden, UK). Farm managers’ opinions were divided about whether electric-powered or water-powered dosing pumps were better, and which brand of water-powered dosing pump was best. Factors influencing their views were: ease of use, reliability, cost, access to reliable mains power, ease of repair, and the size of the building the pump was required to service. One manager had multiple water-powered dosing pumps, each dedicated to a particular antibiotic. Another manager used a water-powered dosing pump for high volume dosing events and an electric-powered dosing pump for low volume dosing events. Some managers using Select dosing pumps were unclear about the maximum pumped output of different models and the maximum water pressure for which each was suitable. Only one manager using a Select dosing pump valued its inbuilt water flow meter. On most managers’ farms, preventative maintenance of dosing pumps was not performed; instead, pumps were repaired when they failed. Only one farm manager regularly calibrated the pumps.

*I’ll take water powered dosing pumps any day. As long as you’ve got water going through that pump, that pump’s going. Your power can go out. Whatever happens, it doesn’t make any difference.*

*On our farm the capacity of water we’re trying to put through the machines means that that machine never stops.*

*(Dosing pump maintenance) is more reactive than proactive because, really, you don’t want to change stuff until it’s worn out.*

### 2.2. Antibiotics and Other Additives Administered to Pigs In-Water

Farm managers administered several different registered antibiotics in-water, as shown in Table 2. Antibiotic use in pigs and other animals in Australia is regulated by a statutory agency, the Australian Pesticides and Veterinary Medicines Authority (APVMA) [14]. Pig industry antibiotic usage patterns are not reported to regulatory authorities. We found that most antibiotics used were of low importance in human medicine, consistent with a previous survey of antibiotic use in the Australian pig industry [15].

**Figure 1:**
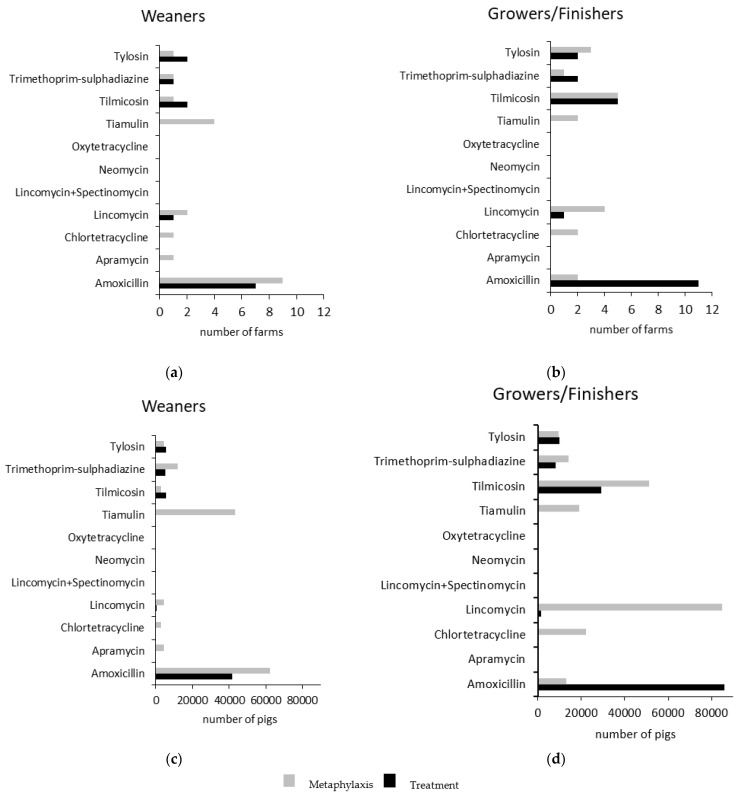
(**a**,**b**) Antibiotics administered in-water to weaner pigs and grower/finisher pigs, respectively, for metaphylaxis and treatment by number of farms. (**c**,**d**) Antibiotics administered in-water to weaner pigs and grower/finisher pigs respectively for metaphylaxis and treatment by number of pigs currently housed on study farms. (25 single-site and multi-site pig farming enterprises, 459,167 growing pigs).

Amoxicillin was the antibiotic most commonly used for treatment of disease outbreaks in weaners and in grower/finishers (Figure 1). It was also the antibiotic most commonly used for metaphylaxis in weaner pigs, followed by tiamulin. In grower/finisher pigs, lincomycin and tilmicosin were most commonly used for metaphylaxis. Simultaneous in-water dosing with more than one antibiotic was practiced on two farms (amoxicillin + apramycin on one farm and tiamulin + tilmicosin on the other).

Drinking water was also used to administer non-antibiotic products to pigs. Organic acid products (most commonly Selko^®^-pH) were administered continuously to weaners as an aid to intestinal health by six managers. However, a further three managers used organic acids periodically to clean pipelines and tanks. One manager used drinking water to administer a proliferative ileitis vaccine (Enterisol), another manager used drinking water to supply electrolytes to newly weaned pigs, and another manager used drinking water to administer sieved weaner manure to maiden gilts as a means of controlling congenital tremors in piglets likely to be caused by a pestivirus.

### 2.3. In-Water Antibiotic Dosing Calculations

All farm managers reported that they strictly followed the prescription provided by their veterinarians to plan each dosing event. They considered in-water antibiotic dosing to be an important task, and so, where practical, only one or two staff members were authorized and trained to perform dosing calculations and conduct dosing events. The prescription for each antibiotic typically detailed the product name and strength to be used, the number of pigs to be medicated and their average bodyweight (kg), the quantity of product to be administered (kg or litres) based on dose rate (mg/kg bodyweight) of the active constituent, the number of dosing events to be conducted on consecutive days, and the applicable meat withholding period. Additional information that was included on the prescription or was left to the farm manager’s discretion included duration and commencement time of each day’s dosing event, the injection ratio of the antibiotic stock solution into the water line, and the corresponding volume of stock solution to be prepared based on an estimated volume of water (litres) that the pigs would use over the dosing period. Many managers were provided with a Microsoft Excel spreadsheet by the veterinarian to assist with these calculations.

*I might have 1000 pigs on arrival, which at say 25 kg, and that gives me 25,000 kg. Then I’ll roughly work those 2500 L they’ll drink. What I’m doing there is then I’m giving them 2.5 kg of Tilmovet into 25 L of water.*

Pig bodyweights used in most dosing calculations were averages estimated from target weight-for-age growth charts. However, on one farm, sentinel pens in each building were weighed regularly and these values were then used in dosing calculations.

*If they’re going to do a 15-week medication, we know they roughly put on 5 kg per week, so by the time they’re 15 weeks they should be around 55 kg. You’re going to have some pigs there that are 40 kg, you might have some that are 60. We do an average over the shed.*

The values for water wastage, as a percentage of total water used by pigs, that were factored into dosing calculations varied widely on managers’ farms, ranging from 0% to 65%, i.e., some farms made no allowance for wastage, assuming that all the antibiotic mixed in the stock solution or header tank would be consumed by pigs. While managers were able to list many factors which could contribute to water wastage, their assumed water wastage rates in buildings fitted with water troughs/bowls varied widely, as did their assumptions for wastage rates in buildings fitted with nipple/bite drinkers. Fixed water wastage values tended to be applied by prescribing veterinarians across buildings on all farms under their supervision, irrespective of the type of drinker being used by the pigs to be dosed and the prevailing seasonal conditions. Only one farm used different water wastage values for summer and winter months.

Most farm managers did not appear to appreciate how the water wastage rate affected the quantity (and therefore cost) of the antibiotic product that must be administered during a dosing event to ensure that the prescribed dose was ingested by pigs on average. They were also not aware of how factoring an inaccurate water wastage value into a dosing calculation could lead to substantial under-dosing or over-dosing.

### 2.4. In-Water Antibiotic Dosing Regimens Used

**Figure A1:**
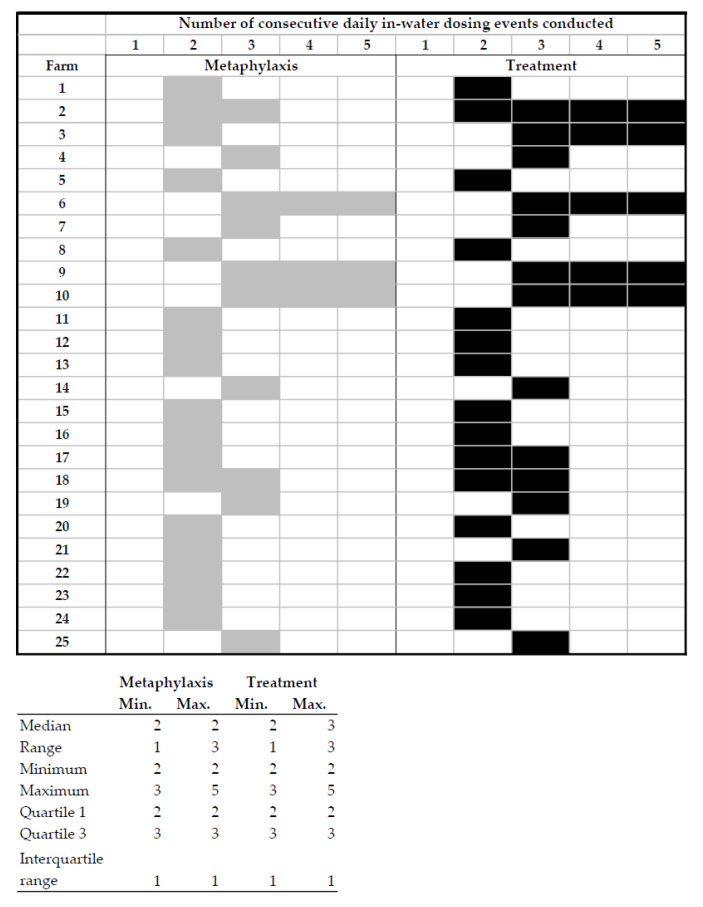
Summary and descriptive statistics for the number of consecutive daily in-water antibiotic dosing events conducted for metaphylaxis and treatment of clinical disease on the 25 pig farms studied.

When medicating pigs, farm managers conducted daily in-water dosing events over two or more consecutive days. The duration of metaphylaxis or treatment of outbreaks ranged from 2 to 5 days. Five managers conducted up to five consecutive daily dosing events if required to resolve a disease outbreak (see Appendix B, Figure A1 for summary and descriptive statistics). Selection of the number of daily in-water dosing events was largely at the manager’s discretion.

*Generally the vet’s script says 3 to 5 days and then we’ll just go off how mortalities are going. If the response is slower we’ll go for 5 days, if it responds quicker we’ll just do the 3 (days).*

**Figure A2:**
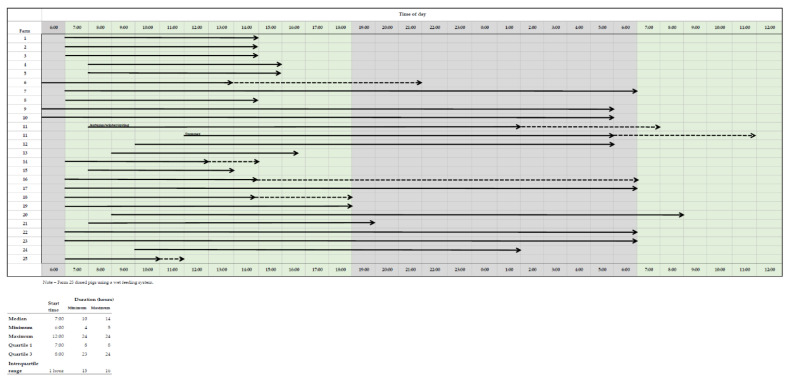
Summary and descriptive statistics for start time and duration of each daily in-water antibiotic dosing event on the 25 pig farms studied.

The time of day at which each daily in-water dosing event was commenced was between 6:00 and 12:00. (Median = 7:00). The duration of each daily dosing event varied widely, from 4 to 24 h. (See Appendix B, Figure A2 for summary and descriptive statistics). Ten farm managers always dosed for a period of ≤8 h, commencing between 6:00 and 9:00. These managers tended to be satisfied that most pigs would ingest sufficient quantities of antibiotic before the end of normal staff working hours (typically 15:00) and wanted to supervise the entire event. Seven farm managers always conducted each dosing event over 24 h, effectively dosing continuously for the chosen number of days.

*Most pigs will have a drink within 8 to 10 h. Every pig has virtually got a hit of amoxicillin.*

*(By dosing for 24 h) we’re picking up the outliers, ones that are a bit scared to go and have a drink, the smaller pigs, ones that possibly are sick and need treatment.*

*If I’m going to only have it for 8 (hours), I guarantee you then that only means they’re going to get it for 5 (hours), and 5 isn’t long enough, not to get the dosage of water. During the day, they’re normally lying there in the afternoon underneath the sprinkler staying cool. They will normally get up and start eating, it might be 7, 8, 9 o’clock when it starts.*

**Figure 2:**
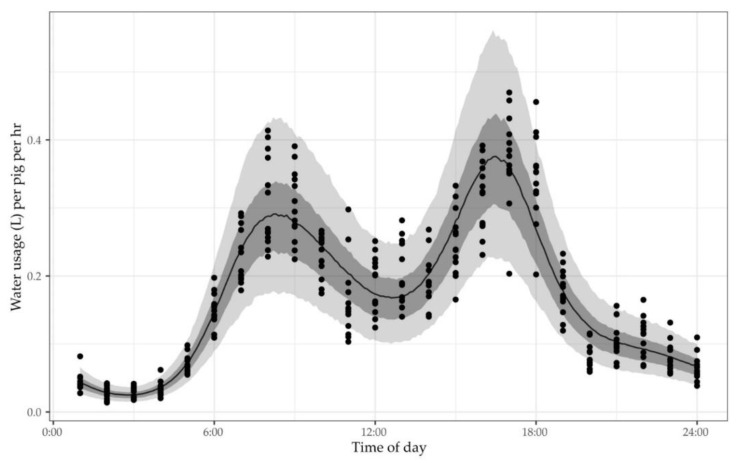
Water usage per pig as a function of time of day over 13 consecutive days (27 August 2020 to 8 September 2020) in a room housing 2150 weaner pigs aged 56 to 69 days of age on Farm 16. The Bayesian hierarchical model for water usage as a smooth function of time of day was generated using the brms package in R. The points are the observations, the solid line the population prediction, and the bands are the 50% and 90% population prediction intervals.

Fourteen farm managers were able to suggest a value for their pigs’ water usage, expressing it in litres per pig per day, percentage of pig bodyweight per day, or litres per day for a given building or the whole farm. When asked when periods of peak water usage occurred each day, managers provided a wide range of responses. Only one manager’s response was based on water usage data collected using a water flow metering system (Farm 16). Analysis of water usage data from one of this manager’s weaner buildings over a 14-day period is provided as an example (Figure 2). These data demonstrated a bimodal pattern of usage with peaks at approximately 8:00 and 16:30.

Most farm managers understood that pigs’ water usage patterns may alter with climatic conditions. However, only one farm (Farm 11) deliberately altered its dosing event commencement time in summer to align it with the high water usage period observed in the afternoon [17]. Seven managers observed changes in water pressure or flow rates during peak demand periods. Some participants managing concrete/slatted floor buildings noted that during extremely hot weather, pigs tended to become restless and squealed, and lay on wet concrete around the drinkers, obstructing other pigs from gaining access.

### 2.5. Preparation of Antibiotic Stock Solutions

The amoxicillin (as amoxicillin trihydrate) products used were generally found by farm managers to be difficult to dissolve at the high concentrations required for dosing pump stock solutions. Sodium carbonate (‘soda ash’) was therefore routinely mixed in water with amoxicillin (usually at a ratio of one part soda ash to three parts amoxicillin by weight) to increase the pH of the stock solution, thereby improving its solubility [18]. Thirteen managers using dosing pumps to administer amoxicillin trihydrate products to pigs, used a range of volumetric injection ratios: 1:33 (three farms), 1:50 (two farms), 1:100 (seven farms) and 1:200 (one farm). Six of these managers dosed pigs over ≤8 h, at injection ratios of 1:33 (two farms), 1:50 (one farms), 1:100 (two farms) or 1:200 (one farm), necessitating the preparation of even more concentrated stock solutions. All managers using amoxicillin trihydrate products at an injection ratio of 1:50 to 1:200 used a magnetic stirrer or small submersible pump to continuously agitate the solution. When discussing the mixability of amoxicillin and other antibiotics, most did not distinguish between the product being in solution or in suspension, unless they observed sediment on the bottom of the container. Recommended protocols for preparing and using stock solutions of specific antibiotic products in proportional dosing pumps were not provided by the manufacturers of antibiotic products or dosing pumps, requiring farm managers (and veterinarians) to develop their own methods.

Lincomycin, the second most commonly used antibiotic, was considered to be very easy to mix and well accepted by pigs. Tiamulin and tilmicosin were also considered easy to use, as they were available in liquid form. Other antibiotics were problematic. Managers reported that tylosin tended to clump when mixed in water and could block filters. It also had an unpleasant taste if the fine powder entered the nose or mouth. Five managers who had used oxytetracycline and chlortetracycline found them particularly difficult to use in dosing pumps. Other antimicrobials that managers had experience using were apramycin (1 farm), trimethoprim/sulphadiazine (5 farms), and neomycin (1 farm).

*I think amoxil is probably the worst (for solubility).*

*Lincomycin mixes well. You mix it up and it stays clear.*

*Lincomycin has a sweet taste, so they just come back for more.*

*Tylosin leaves a horrible taste in your mouth, the dust.*

### 2.6. Frequency of Metaphylactic In-Water Dosing by Phase of Production

**Figure 3:**
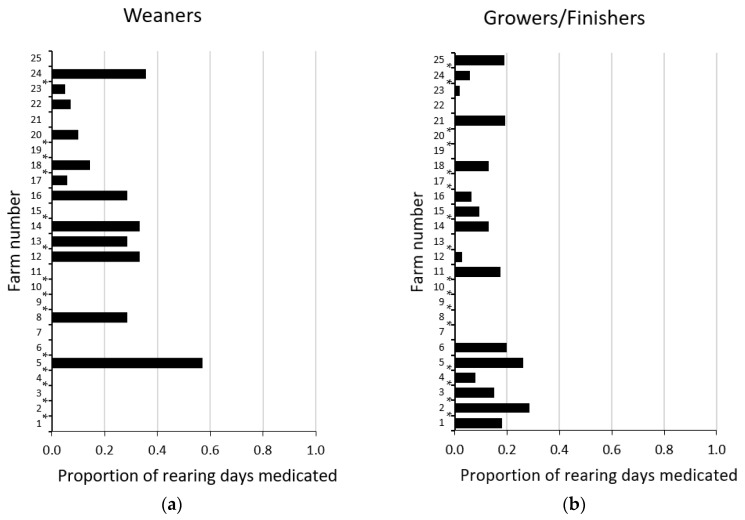
(**a**) Proportion of total rearing days on which one or more antibiotics were administered in-water to weaner pigs for metaphylaxis. (**b**) Proportion of total rearing days on which one or more antibiotics were administered in-water to grower/finisher pigs for metaphylaxis. (25 single-site and multi-site pig farming enterprises, 459,167 growing pigs). Descriptive statistics for weaners: median, 0.00; range, 0.00–0.57; Quartile (Q) 1, 0.00; Q3, 0.29; interquartile range (IQR), 0.29. Descriptive statistics for grower/finishers: median, 0.06; range, 0.00–0.29; Q1, 0.00; Q3, 0.18; IQR: 0.18. Note: * symbol indicates that one or more antibiotics were also administered in-feed to pigs on any rearing days.

There was wide variation in the proportions of total days in the weaner and grower/finisher phases on which pigs on each farm were administered antibiotics in-water for metaphylaxis (Figure 3). Twelve of the 25 farm managers surveyed medicated weaner pigs in-water for metaphylaxis; the other 13 managers only did so for treatment of disease outbreaks. Sixteen managers medicated grower/finisher pigs in-water for metaphylaxis; the other nine managers only used in-water medication for treatment of outbreaks. The highest user of in-water antibiotics per pig (Farm 5) dosed weaner pigs four days per week (two days with amoxicillin followed by two days with chlortetracycline) and dosed grower/finisher pigs for two days per week (alternating amoxicillin and chlortetracycline). There were no significant relationships between the frequency of in-water antibiotic dosing for metaphylaxis in the weaner and grower/finisher phases and (1) the number of weaner and grower/finisher pigs accommodated on farm; (2) type of building (shelters with straw-floor pens vs. conventional buildings with solid/slatted/mesh-floored pens); or (3) production flow (all-in-all-out vs. continuous).

Although continuous in-feed administration of antibiotics is legally permitted in Australia, on 11 managers’ farms, antibiotics were not administered to weaners in feed, and on 10 farms they were not administered to grower/finishers in feed. Some other farms had actively scaled back in-feed administration of antibiotics to pigs or intended to do so. Two managers were setting up dosing equipment in buildings not yet equipped for water medication to make this possible. One manager was proud that, having ceased in-feed medication some years ago, their farm now needed to use very little in-water medication to manage herd health.

*About five years ago the company decided to do no feed medication whatsoever. Now I’m hardly doing any water medication.*

### 2.7. Farm Managers’ Views on Successful In-Water Antibiotic Dosing

Farm managers’ views on successful in-water antibiotic dosing were formed largely by their experiences during disease outbreaks treating sick pigs. Disease outbreaks were a source of considerable concern for managers and their staff, as they were difficult to predict and tended to occur suddenly. Managers judged the success of in-water dosing primarily on the speed with which morbidity and mortalities within a group of sick pigs were reduced after dosing commenced. The frequency of disease outbreaks, pig growth rates, and the uniformity of pig size and body weight were also considered.

During the interviews, farm managers were asked to rate, on a 10-point ordinal scale, how satisfied they were (with 1 being not satisfied at all, and 10 being completely satisfied) that the majority of growing pigs in each building that were water medicated had ingested the dose of antibiotic prescribed by the veterinarian. Twenty managers provided a score. The median score was 7.5 (range, 4–9.5). One manager gave a lower score for dosing to treat pigs in outbreaks (5/10) than for metaphylaxis (7/10) as he felt he had less control in outbreak situations.

Many farm managers did not readily relate the antibiotic dose prescribed by the veterinarian (expressed in mg antibiotic/kg pig bodyweight) to a quantity of antibiotic that needed to be ingested by each pig over a daily in-water dosing event. Managers were not aware that the inhibitory actions of antibiotics differ, with some being time-dependent, some being concentration-dependent, and some being both time and concentration-dependent. Several managers held the view that pigs only needed to ingest some antibiotic during the dosing event for it to be effective in controlling or curing disease.

*The pig only has to drink a litre of water during that six-hour period to get its full medication. It doesn’t matter if it gets that in one mouthful or goes back several times during the day and has a two or three goes at it.*

However, one manager demonstrated an understanding of the sources of variation in systemic exposure of pigs within a group to the antibiotic and why, in a disease outbreak in a building, some pigs would die despite in-water dosing with an appropriate antibiotic:

*There’s 1000 pigs in the shed. Let’s say 900 of them got a drink. Out of that 900 let’s say 600 got the right volume. Out of that 600, let’s say 200 of them didn’t absorb it and it didn’t have the right effect.*

Farm managers suggested a number of factors that could influence the consistency of the dose of antibiotic ingested by pigs in a group during an in-water dosing event. These included:

- Changes in the concentration of the antibiotic in the water over time
- The duration of the dosing period
- Lags in delivery of antibiotic to drinkers in pens further from the dosing pump or header tank
- Variation in the volume of medicated water consumed by pigs due to:
  - Some weanling pigs being slow to start eating and drinking after placement
  - Large pigs consuming more water than smaller pigs
  - Healthy pigs consuming more water than sick pigs
  - Dominant pigs consuming more water than subordinate pigs
  - Pigs’ ease of access to drinkers in the pen (the number of drinkers per pen and/or the position of the drinkers in the pen)
  - Differences in the palatability of different antibiotic products

- Differences in environmental conditions in different sections of building—e.g., cooler south side vs. warmer northern side, due to the tracking of the sun
- The design of the building—the building shape and dimensions, the distances pigs in each pen must walk to feeders and drinkers

In interview discussions with farm managers on many aspects of in-water antibiotic dosing and the in-water and in-feed medication programmes in use on managers’ farms, concerns about antibiotic resistance and the need to preserve the potency of antibiotics through good antibiotic stewardship were not raised by any participants. Nevertheless, all managers reported that a reduction in antibiotic use was a worthy objective for their farm and the pig industry as a whole.

## 3. Discussion

There were two main findings from our study: (1) in-water antibiotic dosing practices varied widely across farms in the antibiotics administered, in the choice and use of dosing equipment, in the methods used for dosing calculations, in the dosing regimens used, in the methods for preparation of antibiotic stock solutions, and in the frequency of metaphylactic dosing of pigs in weaner and grower/finisher phases; and (2) with insufficient measured data and understanding of pharmacology and population pharmacometrics, farm managers were unable to assess the effectiveness of dosing events, optimize in-water dosing calculations and regimens, and control major sources of between-animal variability in systemic exposure of pigs to antibiotics.

### 3.1. Sub-Optimal In-Water Antibiotic Dosing Practices That May Have Contributed to Many Pigs Not Having Sufficient Exposure to Antibiotic

For in-water dosing to successfully reduce pathogen load and achieve high clinical efficacy while minimizing selection and propagation of resistant pathogens in each pig, the antibiotic concentration at the site of infection needs to rise rapidly above the minimum inhibitory concentration (MIC) and attain a target value for the pharmacokinetic/pharmacodynamic (PK/PD) index appropriate for the antibiotic based on its inhibitory action [19]. For example, for amoxicillin (a time-dependent antibiotic), the PK/PD target should be >40% of time 24 h > MIC [20]. On many farms surveyed, the probability that most pigs (e.g., 90%) in a group medicated by in-water dosing would have this level of systemic exposure is likely to be reduced, given the frequency of sub-optimal dosing practices.

Many farm managers who participated in the study used amoxicillin trihydrate and some used trimethoprim-sulphadiazine or chlortetracycline hydrochloride for metaphylactic and/or treatment dosing. Several managers described challenges in dissolving these products in dosing pump stock solution containers or header tanks. This was unsurprising, as these three antimicrobials have much lower solubilities in water than other commonly used antimicrobials [21]. Antimicrobials must be fully dissolved and remain in solution at close to the target concentration for the entire dosing period if the prescribed dose is to be ingested, absorbed, and distributed within each pig [22]. Shorter dosing periods and/or low injection ratios require the amoxicillin stock solution to be very highly concentrated, far in excess of amoxicillin’s solubility threshold, so it is likely that those managers who chose to use a lower injection ratio (e.g., 1:100 or 1:200) and/or a shorter dosing period (≤ 8 h) injected substantial quantities of suspended amoxicillin particles (rather than amoxicillin in solution) into the water pipeline early in the dosing event and thus under-dosed many pigs, even though they used sodium carbonate to improve its solubility and continuous agitated the stock solution. [2,21].

On those farms where dosing pumps were located substantial distances from the buildings they served, the transit time for an antibiotic from the dosing pump to the drinkers may be considerable. This time may provide an opportunity for substantial degradation of the antibiotic if it is exposed to factors that adversely affect its stability over time, such as low water pH, high water hardness, metal piping, the presence of chlorine, metal ions, pH modifiers, and another antibiotic with which it reacts [23,24,25]. On those farms where managers did not perform preventative maintenance on dosing pumps, one would expect failures to have occurred more often during dosing events, resulting in many (or all) pigs not having the required exposure to the antibiotic.

While the solubility thresholds of commonly used antibiotics are not exceeded when solutions are prepared for dosing using header tanks, some variation in the concentration of an antibiotic held in a header tank may occur over time if the solution is not continuously agitated [26]. The three farm managers using header tanks who dosed with each tank’s inlet valve left open would be very unlikely to achieve PK/PD targets, as this practice results in a progressive dilution over the dosing period and thus a decline in the concentration of the antibiotic in the water supplied to the pigs.

The water wastage values used by farm managers (and veterinarians) in their dosing calculations are likely to be inaccurate, as managers lacked access to systems to measure these values in farm buildings prior to dosing events. If the water wastage rate was under-estimated, most (or all) pigs would be under-dosed and therefore not have the required exposure to the antibiotic. Water wastage by pigs has been found in experimental studies to vary widely, from 9% to 60% of total water usage, depending on a range of factors, including water flow rates, drinker design and position, room temperature, levels of competition between pigs, diet, and water quality [27,28,29,30].

In our study, the dosing regimens used by farm managers varied widely in commencement time and duration. The number of consecutive days over which each series of in-water dosing events was conducted also varied. Most managers understood that the dose of antibiotic ingested by pigs was a function of the concentration of antibiotic in the water and the volume of water consumed by each pig hour-by-hour. However, they appeared to not understand that matching commencement of a daily dosing event with the beginning of a period of moderate to high water consumption would result in high hourly rates of antibiotic ingestion by pigs over the first few hours, leading to a more rapid rise in the plasma concentration of the antibiotic and earlier attainment of the PK-PD target for the antibiotic. This approach is consistent with the ‘front-loaded’ dosing regimens used in human critical care medicine, in which a ‘loading dose’ is administered to a patient prior to continuous intravenous infusion in order to reach the desired PK/PD target more rapidly [31,32,33]. An added advantage of this approach is that it helps to minimize the length of time that the plasma concentration of antibiotic lies in the ‘mutant selection window’ just above the MIC, thereby reducing selection for and propagation of resistant pathogens [19,34,35]. We are not aware of any commercially available proportional dosing pumps that can automatically administer an initial loading dose. However, this may be advantageous.

The wide range of responses about the periods of peak water usage each day was consistent with recent studies showing that pig water consumption patterns vary widely between animals and within animals over time [17,29,36,37]. Access to a water flow metering system, allowing the average water usage patterns of a group of pigs to be determined for several days prior to each dosing event, would enable farm managers (and veterinarians) to select the optimal time of the day to commence dosing. This could particularly help those managers conducting in-water dosing events over short periods, i.e., ≤8 h, and those administering low metaphylactic doses over 24 h, to increase the probability that most pigs attain the PK/PD target for the antibiotic (a brief summary of how specific in-water dosing practices would be expected to impact on systemic exposure to an antibiotic is available online in Supplementary Materials).

There is little evidence to guide decisions about the optimal duration of antibiotic therapy in humans or other animals, or specifically in pigs. Past recommendations were largely arbitrary. While human studies comparing morbidity and mortality after shorter or longer courses of antibiotic therapy have yielded inconsistent results, a recent systematic review concluded that reductions in the duration of antibiotic therapy could play an important role in antibiotic stewardship and was feasible for the treatment of many human infectious diseases [38]. Further studies are required in pigs to develop guidelines for the duration of dosing.

Several studies have investigated patterns of antibiotic use in pig production across pig producing countries [15,39,40,41,42,43,44,45,46]. However, this is the first large-scale study that has specifically explored use of in-water antibiotics on commercial pig farms. Without knowledge of the antibiotic dose rate (mg/kg bodyweight) used in each dosing event, we were unable to quantify in-water antibiotic use as ‘treatment incidence’ (TI) based on the number of used daily doses (UDD_pig_) [44]. However, the large variation in the frequency of metaphylactic in-water dosing between farms in our study is consistent with recent studies of oral antibiotic use on pig farms in Australia and other countries [3,15,44,46,47]. On the farms participating in our study, antibiotics were used with higher frequency in the grower/finisher phase than in the weaner phase. This contrasts with other studies [41,47,48,49]. Farm managers’ apparent lack of awareness and concern about antibiotic resistance is consistent with studies in Canada, the UK, and Europe in which pig farmers were found to be more concerned about financial matters and managing herd health than antibiotic resistance [50]. It may also reflect a view among farm managers that managing antibiotic resistance is the responsibility of veterinarians.

### 3.2. Consequences of Sub-Optimal In-Water Antibiotic Dosing Practices on Pig Farms

**Figure 4:**
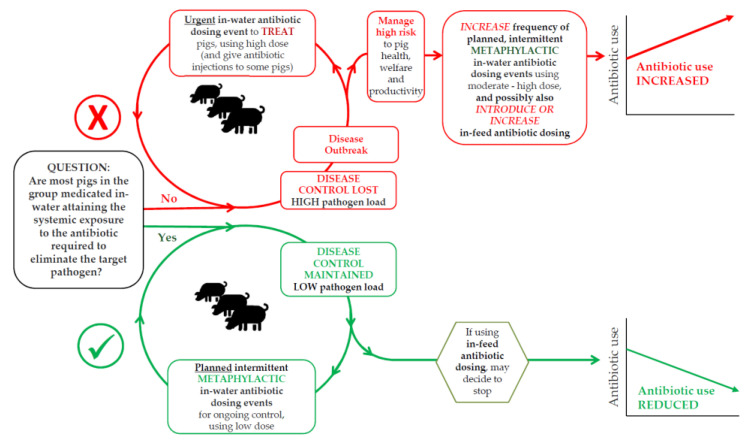
Schematic showing possible pathways resulting in increased antibiotic use (red) or reduced antibiotic use (green) in pigs, based on whether most pigs in the medicated group have sufficient systemic exposure to the antibiotic to eliminate the target pathogen.

If sub-optimal in-water antibiotic dosing practices, as found on many farms surveyed, led to most pigs in a group medicated not having sufficient systemic exposure to the antibiotic to eliminate (or substantially reduce the quantity of) the target pathogen, it is plausible that the pathogen load in the pigs would remain high and a disease outbreak may occur, necessitating urgent in-water antibiotic dosing at a high dose rate [5,35] (Figure 4). If this dosing also failed to eliminate or substantially reduce the pathogen load in most pigs, then there may be a repeating cycle of disease outbreaks requiring urgent in-water treatment dosing. Antibiotic use (and cost) per pig produced on these farms may be further increased if, through a desire by farm managers and veterinarians to manage risk to pig health, welfare, and productivity, metaphylactic in-water dosing is conducted more frequently using moderate to high doses, and/or in-feed medication is introduced, or increased. Increased antibiotic use may have further consequences, including increased selection for and propagation of resistant pathogens and increased dissemination of antibiotics in effluent to the environment. If a farm’s in-water dosing practices are such that many pigs in a group do not ingest a sufficient dose of antibiotic during an in-water dosing event, then many pigs may also not ingest a sufficient quantity of a non-antibiotic additive with dose-dependent efficacy if it were administered in water. This warrants future investigation.

### 3.3. Limitation of the Study

The survey successfully integrated qualitative and quantitative data on the in-water antibiotic dosing practices of a sample of Australian pig farm managers. However, the survey has limitations. Our sample was not randomly selected from the population of Australian pig farm managers. This is explained in Materials and Methods. The survey was focused on pig farm managers’ in-water dosing practices and physical characteristics of each farm relevant to in-water dosing. We did not survey veterinarians to collect data on pigs’ health status and performance, the prescribing behaviour of veterinarians, or the efficacy of the medication programmes implemented. Our survey should be considered as a first step in gaining a detailed understanding of in-water antibiotic dosing practices used by pig farm managers. Practices may differ in each country. Further research is therefore needed to compare our findings on the in-water antibiotic dosing practices of pig farm managers with those in other countries.

### 3.4. Conclusions

There is considerable scope to improve in-water antibiotic dosing practices on commercial pig farms, and thereby increase the effectiveness of in-water dosing and reduce antibiotic use (and cost) on farms. To enable farm managers (and veterinarians) to achieve these outcomes, they would require access to (1) on-farm measuring systems that provide easily interpretable data on the water wastage and daily water usage pattern of each group of pigs being dosed; and (2) technical guidelines and training on in-water antibiotic dosing based on key principles of antibiotic pharmacology and population pharmacometrics. Development and extension of the technical guidelines and training programs would need to be led by industry, supported by commercial pig enterprises, and involve a multi-disciplinary development and training team of pig veterinarians, pharmacologists, and other professionals with relevant expertise. With these measurement systems, technical guidelines and training in place, veterinarians would be better able to work with farm managers in designing and conducting in-water dosing and perform regular audits of farms’ in-water dosing systems and practices.

## 4. Materials and Methods

Farm managers were recruited for this study using a purposive sampling method that aimed to obtain a sample population of farm managers of medium to large single-site and multi-site pig farming enterprises across Australia. To be eligible to participate in the study, a person was required to be a pig farm manager responsible for management of the water system and in-water antibiotic dosing of growing pigs. Their farm must have operated for at least six months, have more than 500 weaner and grower pigs, participated in the Australian Pork Industry Quality Assurance Program, reared growing pigs indoors (in concrete-floored rooms/sheds or in straw-floored shelters), and water medicated weaner and/or grower/finisher pigs with antibiotics for metaphylaxis and treatment of bacterial diseases. The cohort of farms included in the study was non-random, but representative of the Australian pig industry, comprising approximately 21% of all growing pigs accommodated in Australia at any time. Each farm manager was contacted directly by telephone and invited to participate in the survey. They were then emailed a detailed handout explaining the study’s aims and design and a consent form for signature and return prior to commencement.

The study used a mixed-methods approach. An initial quantitative phase comprised an online questionnaire. Once completed, a qualitative phase followed, comprising a one-on-one semi-structured interview. The online questionnaire and the interview were designed to each be completed by participants in less than 45 min. The online questionnaire (available online in pdf format in Supplementary Materials) was designed to provide an understanding of variability across farms regarding the features of the buildings in which growing pigs were reared, drinking water supply systems, and water medication dosing systems and programmes. It used recommended design features to make it more effective and user-friendly [51,52]. The questionnaire was created and managed in REDCap (Research Electronic Data Capture), a secure web-based application for building and managing online surveys (Vanderbilt University, Nashville, Tennessee, USA). Participants were required to respond to all questions and asked to complete the questionnaire within two weeks of receipt using a web-link. The most demanding part of the questionnaire was that seeking characteristics of two weaner buildings and two grower/finisher buildings on the farm, as respondents needed to consult farm records and make some measurements. This part was therefore positioned near the beginning of the questionnaire. This appeared to assist users. Thorough pre-testing was conducted with colleagues and farm managers and refinements were made to improve the questionnaire’s clarity and ease of use before it was deployed.

Each farm manager was interviewed within four weeks of completing the online questionnaire. Each interview was conducted using an interview guide based on a review of the literature and previous discussions by the lead author (S.L.) with farm managers and veterinarians. The interview guide (available online in Supplementary Materials) comprised mostly open questions to facilitate discussion of the design and function of the farm’s drinking water distribution system, the pigs’ daily water usage patterns, the provision of water to pigs in specific buildings, and the in-water medication dosing process used. Two pilot interviews were conducted, and the interview guide was reviewed and revised prior to use. The changes made were mainly to terminology.

Quantitative data collected from each farm manager in the online questionnaire were used in the interview to tailor questions to each participant and encouraged richer, more detailed responses from participants. Every effort was made not to direct or influence the participant’s responses. All interviews were conducted by S.L. Interviews were initially conducted face-to-face on the farm at a time convenient for the participant. However, due to logistical challenges and more stringent biosecurity measures imposed by farms during the study to manage the risk of African swine fever, later interviews were conducted by telephone. Each interview was recorded on a digital recorder (audio only) with the permission of each participant to facilitate later qualitative analysis.

Questionnaire responses captured in REDCap from each participant were exported into Excel, de-identified, and then subjected to statistical analysis in Excel and R. Interviews were transcribed verbatim from audio recordings into text documents and de-identified. Transcripts were then entered into the qualitative data analysis software package NVivo version 12 (QSR International Pty. Ltd., Melbourne, Victoria, Australia) and openly coded and analysed by the interviewer using qualitative data analysis principles and thematic analysis [53]. Coding of transcripts was done manually. This was an iterative process, with nodes being refined as more data were coded. The final coding framework used in NVivo is available online in Supplementary Materials. Sentiment analysis was performed in NVivo using automatic coding. Selected comments made by participants in the interviews that the lead author found illustrative were included in the Results section.

## Figures and Tables

**Table 1:** In-water antibiotic dosing equipment used by farm managers on 25 single-site and multi-site pig farming enterprises.

Dosing Equipment Item	Weaner Buildings (No. Farms = 23)	Grower/Finisher Buildings (No. Farms = 24)
Header tank for batch mixing	3 (12%)	1 (4%)
Water-powered proportional dosing pump	8 (32%)	10 (40%)
Electric-powered proportional dosing pump	10 (40%)	9 (36%)
Water-powered and electric-powered proportional dosing pumps	1 (4%) ^a^	1 (4%) ^a^
Header tank for batch mixing plus electric-powered proportional dosing pump	1 (4%) ^b^	2 (8%) ^b,c^
Dispenser into liquid-feed system	0 (0%)	1 (4%)

^a^ Uses different pumps for different antibiotics; ^b^ Currently changing buildings from header tanks to electric-powered dosing pumps; ^c^ Uses header tanks and electric-powered dosing pumps together for treating outbreaks.

**Table 2:** Antibiotics currently and recently administered in-water to pigs on 25 single-site and multi-site pig farming enterprises.

Antibiotic	Product Form	Class	Importance in Human Medicine ^a^
Amoxicillin	Powder	β-lactam	Low
Apramycin	Powder	Aminoglycoside	Medium
Chlortetracycline	Powder	Tetracycline	Low
Lincomycin	Powder	Lincosamide	Medium
Lincomycin + Spectinomycin	Powder	Lincosamide + Aminocyclitol	Medium
Neomycin	Powder	Aminoglycoside	Medium
Oxytetracycline	Powder	Tetracycline	Low
Tiamulin	Liquid	Pleuromutilin	Low NHU ^b^
Tilmicosin	Liquid	Macrolide	Low
Trimethoprim-sulphadiazine	Powder	Dihydrofolate reductase inhibitor + sulfonamide	Medium
Tylosin	Powder	Macrolide	Low

^a^ Based on list of the Australian Strategic and Technical Advisory Group (ASTAG), 2018 [16]; ^b^ No human use (NHU) of the antibiotic class in Australia.

## Data Availability

The data presented in this study are available on request from the corresponding author. The data are not publicly available due to conditions of the ethics agreement.

## Supplementary Materials

The following are available online at https://www.mdpi.com/2079-6382/10/2/169/s1: S1: Online questionnaire created and managed in REDCap (pdf version); S2: Semi-structured interview guide; S3: Final coding framework [NVivo] used to analyse interview transcripts; and S4: Summary table ‘Specific in-water dosing practices, their consequences and the expected impact on pigs’ systemic exposure to an antibiotic administered through their drinking water’.

## Appendix A

**Table A1:** Demographics of the 25 pig farm managers who participated in the study.

Characteristic	Study Participants N
Gender:	
Male	23
Female	2
Age:	
<25	0
25–34	1
35–44	5
45–54	9
>55	10
Years working in pig industry:	
<2	0
2–5	1
6–10	3
>10	21
Years managing current farm:	
<2	6
2–5	3
6–10	4
>10	12

**Table A2:** Characteristics of the 25 pig farms studied.

Characteristic	Study Farms %
Location [state of Australia]:	
South Australia	36%
Victoria	32%
New South Wales	24%
Queensland	8%
Animals on farm:	
Sows, boars and growing pigs	48%
Growing pigs only	52%
Single- or multiple-site configuration:	
Single	72%
Multiple	28%
Weaner pig buildings described in questionnaire:	
Type:	
Solid/slatted/mesh-floored pens in conventional buildings	61%
Straw-floor pens in eco-shelters	39%
Age of buildings:	
<2 years	22%
3–10 years	0%
11–20 years	34%
20 years	44%
Grower/finisher pig buildings described in questionnaire:	
Type:	
Solid/slatted/mesh-floored pens in conventional buildings	30
Straw-floor pens in eco-shelters	17
Age of buildings:	
<2 years	12%
3–10 years	6%
11–20 years	30%
20 years	52%
Pig flow in weaner buildings	
All-in-all-out by room or building	94%
Continuous flow	6%
Pig flow in grower/finisher buildings	
All-in-all-out by room or building	78%
Continuous flow	22%
