# Supplementary material for: In-Water Antibiotic Dosing Practices on Pig Farms
Source: Antibiotics (Basel). 2021 Feb 8;10(2):169. doi: 10.3390/antibiotics10020169 (PMC7915319; doi:10.3390/antibiotics10020169)
Supplement: Supplementary file 1 [file antibiotics-10-00169-s001.zip › S1 - Online questionnaire (REDCap).pdf]

Pig Water Medication Questionnaire

Please complete this questionnaire.

Thank you very much for taking the time to complete this questionnaire. The information you provide will be used to develop tools to help pig producers and veterinarians optimise dosing of groups of pigs on farms with antibiotics and other additives via drinking water for disease control and treatment.

This questionnaire has 4 main parts. It should take you no more than 45 minutes to complete.

As explained in the Consent Form, all the information you provide will remain confidential.

You do not have to complete the questionnaire in one session. Click the 'Save & Return Later' button on the survey page to save your progress and return where you left off to complete the survey any time in the future.

Many thanks,

Dr. Steve Little

PhD student

Faculty of Veterinary and Agricultural Sciences

The University of Melbourne

1. GENERAL INFORMATION

Firstly, please provide some general information about you and your farm.

Name: ______________________________

Mobile number: ______________________________

Farm name: ______________________________

Farm road address: ______________________________

Management position on farm: ______________________________

How many years have you worked in the pig industry?
- ◯ Less than 2 years
- ◯ 2 to 5 years
- ◯ 6 to 10 years
- ◯ More than 10 years

How many years have you worked on this particular farm?
- ◯ Less than 2 years
- ◯ 2 to 5 years
- ◯ 6 to 10 years
- ◯ More than 10 years

How old are you?
- ◯ 18 to 24
- ◯ 25 to 34
- ◯ 35 to 44
- ◯ 45 to 54
- ◯ 55 to 64
- ◯ 65 or over
- ◯ Decline to answer

Farm is:
- ◯ Farrow-to-finish
- ◯ Growing pigs only

Farm production system:
- ◯ All-in-all-out by room or building
- ◯ Continuous flow
- ◯ All-in-all-out in some rooms / buildings, continuous flow in others

Pig breed: ________________________________

Suckling pigs - current number on farm: ________________________________

Suckling pigs - no. days in this phase: ________________________________

Weaner pigs - current number on farm: ________________________________

Weaner pigs - no. days in this phase: ________________________________

Grower-finisher pigs - current number on farm: ________________________________

Grower-finisher pigs - no. days in this phase: ________________________________

Sows - current number on farm: ________________________________

2A. FARM'S WEANER BUILDINGS

Please select two (2) weaner buildings on the farm in which pigs are water medicated.
If possible, select 2 buildings that are different in design to each other.

FIRST WEANER BUILDING:

Building's name: ________________________________

Building's age:
- ◯ Less than 2 years
- ◯ 3 to 10 years
- ◯ 11 to 20 years
- ◯ More than 20 years

Number of rooms: ________________________________

Number of pens per room: ________________________________

Number of pigs per pen: ________________________________

Approximate pen length (metres): ________________________________

Approximate pen width (metres): ________________________________

Number of feeders per pen: ________________________________

Feeder type:
- ◯ Dry
- ◯ Wet/dry

Number of drinkers per pen: ________________________________

Drinker type (select one or more):
- ☐ Nipple
- ☐ Bite
- ☐ Bowl
- ☐ Trough
- ☐ Nipple + bite
- ☐ Nipple + bowl
- ☐ Nipple + trough
- ☐ Bite + trough

Pen floor type (select one or more):
- ☐ Full mesh or slat
- ☐ Partial mesh or slat
- ☐ Solid
- ☐ Straw
- ☐ Other

Pigs' sex (select one or more):
- ☐ Boar
- ☐ Gilt
- ☐ Barrow

Pigs' average age at entry (days): ________________________________

Pigs' average bodyweight at entry (kg): ________________________________

Pigs' average age at exit (weeks): ________________________________

Pigs' average bodyweight at exit (kg): ________________________________

Average days each pig is in building (days): ________________________________

All-in-all-out by room or building?
- ◯ Yes
- ◯ No

Ventilation system in building:
- ◯ Natural
- ◯ Mechanically assisted (fans)

Is the building's ceiling insulated?
- ◯ Yes
- ◯ No

Number of other weaner buildings on the farm like this one: ______________________________

SECOND WEANER BUILDING:

Building's name: __________________________________

Building's age:
- ◯ Less than 2 years
- ◯ 3 to 10 years
- ◯ 11 to 20 years
- ◯ More than 20 years

Number of rooms: __________________________________

Number of pens per room: __________________________________

Number of pigs per pen: __________________________________

Approximate pen length (metres): __________________________________

Approximate pen width (metres): __________________________________

Number of feeders per pen: __________________________________

Feeder type:
- ◯ Dry
- ◯ Wet/dry

Number of drinkers per pen: __________________________________

Drinker type (select one or more):
- ☐ Nipple
- ☐ Bite
- ☐ Bowl
- ☐ Trough
- ☐ Nipple + bite
- ☐ Nipple + bowl
- ☐ Nipple + trough
- ☐ Bite + trough

Pen floor type (select one or more):
- ☐ Full mesh or slat
- ☐ Partial mesh or slat
- ☐ Solid
- ☐ Straw
- ☐ Other

Pigs' sex (select one or more):
- ☐ Boar
- ☐ Gilt
- ☐ Barrow

Pigs' average age at entry (days): __________________________________

Pigs' average bodyweight at entry (kg): __________________________________

Pigs' average age at exit (weeks): __________________________________

Pigs' average bodyweight at exit (kg): __________________________________

Average days each pig is in building (days): __________________________________

All-in-all-out by room or building?
- ◯ Yes
- ◯ No

Ventilation system in building:
- ◯ Natural
- ◯ Mechanically assisted (fans)

Is the building's ceiling insulated?
- ◯ Yes
- ◯ No

Number of other weaner buildings on the farm like this one: ________________________________

2B. FARM'S GROWER/FINISHER BUILDINGS

Please select two (2) grower/finisher buildings on the farm in which pigs are water medicated.

If possible, select 2 buildings that are different in design to each other.

FIRST GROWER/FINISHER BUILDING:

Building's name: ______________________

Building's age:
- ◯ Less than 2 years
- ◯ 3 to 10 years
- ◯ 11 to 20 years
- ◯ More than 20 years

Number of rooms: ______________________

Number of pens per room: ______________________

Number of pigs per pen: ______________________

Approximate pen length (metres): ______________________

Approximate pen width (metres): ______________________

Number of feeders per pen: ______________________

Feeder type:
- ◯ Dry
- ◯ Wet/dry

Number of drinkers per pen: ______________________

Drinker type (select one or more):
- ☐ Nipple
- ☐ Bite
- ☐ Bowl
- ☐ Trough
- ☐ Nipple + bite
- ☐ Nipple + bowl
- ☐ Nipple + trough
- ☐ Bite + trough

Pen floor type (select one or more):
- ☐ Full mesh or slat
- ☐ Partial mesh or slat
- ☐ Solid
- ☐ Straw
- ☐ Other

Pigs' sex (select one or more):
- ☐ Boar
- ☐ Gilt
- ☐ Barrow

Pigs' average age at entry (weeks): ______________________

Pigs' average bodyweight at entry (kg): ______________________

Pigs' average age at exit (weeks): ______________________

Pigs' average bodyweight at exit (kg): ______________________

Average days each pig is in building (days): __________________________________

All-in-all-out by room or building?
- ◯ Yes
- ◯ No

Ventilation system in building:
- ◯ Natural
- ◯ Mechanically assisted (fans)

Is the building's ceiling insulated?
- ◯ Yes
- ◯ No

Number of other grower/finisher buildings on the farm like this one: __________________________________

SECOND GROWER/FINISHER BUILDING:

Building's name: ________________________________

Building's age:
- ◯ Less than 2 years
- ◯ 3 to 10 years
- ◯ 11 to 20 years
- ◯ More than 20 years

Number of rooms: ________________________________

Number of pens per room: ________________________________

Number of pigs per pen: ________________________________

Approximate pen length (metres): ________________________________

Approximate pen width (metres): ________________________________

Number of feeders per pen: ________________________________

Feeder type:
- ◯ Dry
- ◯ Wet/dry

Number of drinkers per pen: ________________________________

Drinker type (select one or more):
- ☐ Nipple
- ☐ Bite
- ☐ Bowl
- ☐ Trough
- ☐ Nipple + bite
- ☐ Nipple + bowl
- ☐ Nipple + trough
- ☐ Bite + trough

Pen floor type (select one or more):
- ☐ Full mesh or slat
- ☐ Partial mesh or slat
- ☐ Solid
- ☐ Straw
- ☐ Other

Pigs' sex (select one or more):
- ☐ Boar
- ☐ Gilt
- ☐ Barrow

Pigs' average age at entry (weeks): ________________________________

Pigs' average bodyweight at entry (kg): ________________________________

Pigs' average age at exit (weeks): ________________________________

Pigs' average bodyweight at exit (kg): ________________________________

Average days each pig is in building (days): ________________________________

All-in-all-out by room or building?
- ◯ Yes
- ◯ No

Ventilation system in building:
- ◯ Natural
- ◯ Mechanically assisted (fans)

Is the building's ceiling insulated?
- ◯ Yes
- ◯ No

Number of other grower/finisher buildings on the farm like this one: ________________________________

3. FARM'S DRINKING WATER SUPPLY SYSTEM

What is the main water source the farm has used over the past 12 months?

- ◯ Town supply
- ◯ Bore
- ◯ River, lake or irrigation channel
- ◯ Farm dam
- ◯ Bore + town supply (shandy)
- ◯ Bore + river, lake or irrigation channel (shandy)
- ◯ Bore + farm dam (shandy)

What other water source(s) has the farm used over the past 12 months?

- ☐ Town supply
- ☐ Bore
- ☐ River, lake or irrigation channel
- ☐ Farm dam
- ☐ Bore + town supply (shandy)
- ☐ Bore + river, lake or irrigation channel (shandy)
- ☐ Bore + farm dam (shandy)

What water storage facilities are on the farm?

- ☐ None
- ☐ Dam
- ☐ Primary tank(s) (covered to prevent contamination)
- ☐ Primary tank(s) (uncovered)
- ☐ Header tank(s) on buildings (covered to prevent contamination)
- ☐ Header tank(s) on buildings (uncovered)
- ☐ Other

You selected Other. Please describe water storage facility.

____

How is water delivered to buildings on the farm?

- ☐ Gravity
- ☐ Pump(s)
- ☐ Mains pressure
- ☐ Other

You selected Other. Please describe how water is delivered to buildings.

____

EXTERNAL main water supply line from source to buildings on the farm

	Straight main pipe with branches to each building	Looped main pipe around buildings
To weaner buildings	☐	☐
To grower/finisher buildings	☐	☐

INTERNAL water supply line in buildings on the farm

	Straight main pipe with lateral branches to rows of pens	Looped main pipe around rows of pens
In weaner buildings	☐	☐
In grower/finisher buildings	☐	☐

Water pipe materials

	White Poly	Black PVC	Metal	Concrete	Other
External pipes to weaner buildings	☐	☐	☐	☐	☐
External pipes to grower/finisher buildings	☐	☐	☐	☐	☐
Internal pipes in weaner buildings	☐	☐	☐	☐	☐
Internal pipes in grower/finisher buildings	☐	☐	☐	☐	☐

Water flow rates from pen drinkers:

Are there any concerns about the water flow rates from pen drinkers to pigs in any buildings on the farm? e.g. flow rates too low, too high, too inconsistent
◯ Yes
◯ No

You selected Yes. Please describe concerns. ________________________________

How often are the water flow rates from pen drinkers in the buildings you described in section 2 checked?
◯ Daily
◯ Weekly
◯ Monthly
◯ Quarterly
◯ 6 monthly
◯ Yearly
◯ Sporadically
◯ Never

What method is used to check water flow rate from pen drinkers?
◯ Visual assessment only
◯ Use finger to assess strength of flow from drinker
◯ Use a graduated measuring jug and timer to measure flow rate in mls/minute
◯ Other

What time of day are the checks usually done?
◯ 12am to 6am
◯ 6am to 12pm
◯ 12pm to 6pm
◯ 6pm to 12am

How many drinkers in each building are checked each time?
◯ All
◯ Many
◯ Some

Have you measured drinker flow rates inside any of the buildings you described in section 2?
◯ Yes
◯ No

FIRST weaner building:

Flow rate - Average (mls/minute) ________________________________

Flow rate - Minimum (mls/minute) ________________________________

Flow rate - Maximum (mls/minute) ________________________________

SECOND weaner building:

Flow rate - Average (mls/minute) ________________________________

Flow rate - Minimum (mls/minute) ________________________________

Flow rate - Maximum (mls/minute) ________________________________

FIRST grower/finisher building:

Flow rate - Average (mls/minute) ________________________________

Flow rate - Minimum (mls/minute) ________________________________

Flow rate - Maximum (mls/minute) ________________________________

SECOND grower/finisher building:

Flow rate - Average (mls/minute) __________________________________

Flow rate - Minimum (mls/minute) __________________________________

Flow rate - Maximum (mls/minute) __________________________________

Water line cleaning and maintenance:

How often is the complete water line (including header tanks, pipes and drinkers) flushed and cleaned/sanitised?

- ◯ Daily
- ◯ Weekly
- ◯ Monthly
- ◯ Quarterly
- ◯ 6 monthly
- ◯ Yearly
- ◯ Between each batch of pigs
- ◯ Sporadically
- ◯ Never

Please describe the method / materials / chemicals used.

____

Are drinkers thoroughly cleaned/sanitised between each batch of pigs?

- ◯ Yes
- ◯ No

Please describe routine repairs and maintenance done to water line, including header tanks, pipes and drinkers (what is done and when).

____

Water quality:

Are there any concerns about the quality of water supplied to pigs on the farm? e.g. its physical appearance, chemical and mineral composition, or microbial contamination

◯ Yes
◯ No

You selected Yes. Please describe concerns.

____

How often is a sample of the farm's drinking water collected and quality tested at a laboratory?

◯ Every 6 months
◯ Every year
◯ Every 2 years
◯ Sporadically
◯ Never

Do you have a copy of the most recent water test report?

◯ Yes
◯ No

Are you willing to share this water test report to assist this research project?
(It would remain confidential)

◯ Yes
◯ No

Is the farm's drinking water routinely treated in any way to improve its quality?

◯ Yes
◯ No

How is the water treated?

☐ Activated carbon filtration
☐ Reverse osmosis (RO)
☐ Ion exchange water softening
☐ Sediment filtration
☐ Distillation
☐ Aeration
☐ De-aeration
☐ Continuous chlorination
☐ Ultraviolet radiation (UV)
☐ Ozonation
☐ Ultra, micro and nano filtration
☐ Other

You selected Other. Please specify.

____

4. FARM'S WATER MEDICATION DOSING SYSTEM AND PROGRAM

How many years has your farm been water medicating pigs?

◯ Less than 2 years
◯ 2 to 5 years
◯ 6 to 10 years
◯ 11 to 20 years
◯ More than 20 years

Type of dosing equipment serving each of the buildings you described in section 2.

	Header tank for batch mixing	Water powered proportional dosing pump e.g. Gator, Dosatron	Electric powered, computer controlled dosing pump e.g. Select
First weaner building	☐	☐	☐
Second weaner building	☐	☐	☐
First grower/finisher building	☐	☐	☐
Second grower/finisher building	☐	☐	☐

Does each pump serve more than one building?
◯ Yes
◯ No

Does each pump have bypass valves to isolate it when not in use?
◯ Yes
◯ No

Does each pump have a filter upstream to trap sediment?
◯ Yes
◯ No

Does each pump have a water flow meter?
◯ Yes
◯ No

Does each pump have a pressure reducer to protect it against pressure surges?
◯ Yes
◯ No

In each of the buildings you described in section 2:

	Water medication is administered to pigs in ALL pens within building	Water medication can be administered to pigs in selected pens within building
First weaner building	○	○
Second weaner building	○	○
First grower/finisher building	○	○
Second grower/finisher building	○	○

What is the SHORTEST distance between a dosing pump and a weaner building on the farm? (metres): ________________________________

What is the LONGEST distance between a dosing pump and a weaner building on the farm? (metres): ________________________________

What is the SHORTEST distance between a dosing pump and a grower/finisher building on the farm? (metres): ________________________________

What is the LONGEST distance between a dosing pump and a grower/finisher building on the farm? (metres): ________________________________

Route by which medications are administered to pigs in the buildings you described in section 2:

	In-water	In-feed	By injection
First and second weaner buildings	☐	☐	☐
First and second grower/finisher buildings	☐	☐	☐

Please list water medications (brand name or active) administered to pigs in the first and second weaner buildings: ________________________________

Please list water medications (brand name or active) administered to pigs in the first and second grower/finisher buildings: ________________________________

Thank you very much for completing the questionnaire.
