# Supplementary material for: In-Water Antibiotic Dosing Practices on Pig Farms
Source: Antibiotics (Basel). 2021 Feb 8;10(2):169. doi: 10.3390/antibiotics10020169 (PMC7915319; doi:10.3390/antibiotics10020169)
Supplement: Supplementary file 1 [file antibiotics-10-00169-s001.zip › S2 - Semi-structured interview guide.pdf]

Interview (semi-structured)
Interview guide

1. Opening of interview

Welcome, name.

Small talk & acclimatization.

Inform interviewee that I am switching on the digital recorder.
"Just letting you know that I'm turning on the recorder now."

Opening statement
"Thank you very much for taking the time to meet with me and do this interview, further to the questionnaire, as part of our research project."

"During this interview today I would like to learn more about your farm's drinking water supply system and how you carry out water medication dosing on your farm."

"Sometimes I might ask you to give an example or an opinion. Feel free to take your time and think about it. Remember, I'm interested in hearing your views and experiences, so there are no right or wrong answers. And you don't have to talk about anything you don't want to. You can stop the interview at any time, take a break, or end the interview altogether – just let me know."

"This interview will take up to one hour. No longer. As mentioned before and on the consent form it is recorded. I would like to emphasize that anything you say will be treated as confidential. This means that your responses will be de-identified so that your identity and details of your property/location will not be known."

"Are you willing to proceed with the interview?"

"Do you have any questions before we begin?"

"OK. Let's proceed!"

2. Farm's drinking water supply system

Water supply system design

"Firstly, just a little more detail further to the questionnaire, regarding the design of your water supply system."

Prompts:

- Over time, to what extent has the farm expanded its pig numbers and the number / size of weaner and grower/finisher buildings?
- How has the water supply system been upgraded as part of this expansion?
- Does the water supply system have some features which limit its ability to supply water to some pigs during periods of peak demand? e.g. during summer and when pressure washing is being done
- What changes (for better or worse) has this expansion (if any) led to regarding provision of water to pigs in pens across all the weaner and grower/finisher buildings? e.g. change in no. pigs per drinker, water pressure and flow rates from drinkers
- Do you have each of the following fittings installed anywhere along the farm's water line between the water source and pen drinkers in each weaner and grower/finisher building? If so, where, and what value does it provide?
    - Backflow prevention valves (check valve, non-return valve)
    - Water pressure regulators
    - Water pressure gauges
    - Water usage meters
    - Pump breakdown alarms

Daily water usage and demand patterns

Prompts:

- How much water per day (litres) do you estimate is used by each pig in the weaner and grower/finisher buildings you described in part 2 of the questionnaire? Have you been able to accurately measure daily water usage in these buildings?
- What proportion of daily water usage by these pigs (as a percentage) do you think is spilt and wasted rather than drunk?
- What do you think are the main factors that influence daily water wastage rates? (List)
- Do levels of water wastage create any concerns or problems on the farm? If so, what?
- At what time(s) of the day do you think peak water demand periods occur in the weaner and grower/finisher buildings you described in part 2 of the questionnaire?
- During these peak water demand periods have you observed:
    - Changes in water pressure and flow rates from drinkers across the buildings? If so, what and why?
    - Changes in pig behaviour? If so, what?
- Can you recall an incident in the past few months or years when the water supply system failed to satisfy some pigs' water demand in any buildings on the farm? What happened?

Provision of water to pigs

Prompts:

- *Refer to respondent's questionnaire (parts 2 and 3) and pose questions regarding:*
    - *how water flow rates from drinkers are regulated (use of pressure regulators, nipple inserts)*
    - *drinker design, number of drinkers per pen, drinker position, height, angle, location in pen*
    - *strengths and limitations of different types of drinkers based on their experience*
- Are there any other challenges / problems / risks when providing water to pigs?

3. Water medication dosing

Weaner and grower/finisher water medication programs

"In part 4 of the questionnaire you listed the medications administered in water to pigs in the weaner and grower/finisher buildings you described in part 2 of the questionnaire over the past 12 months.
These medications were ……….."

"Now, for each of the water medication products currently being used in weaners, and used in grower/finishers, can you please tell me:

- Is the product administered at a set age / bodyweight or regular time interval (e.g. every week, month) for disease prevention/control, or only when disease is detected?
- What proportion of all pigs accommodated in each building were administered this product? (Calculate % animals)
- For what proportion of their total days in the building were the pigs administered this product? (Calculate % animal days)

Water medication dosing process

"Now, in this final part of this interview, I would like to discuss the steps involved in carrying out a water medication dosing 'event' on your farm."

Prompts:
Step 1: Calculating the dosage

- How is the concentration of a given medication product in water (grams or mls product per litre water) for given group of pigs calculated?
    - Are there any challenges / risks / problems that you face?
    - What written dosage directions are provided by your veterinarian? How useful are these?
    - When making dosage calculations for a water medication dosing 'event':
        - How are the pig bodyweights estimated / calculated?
        - What values for pigs' daily water intake at different bodyweights are used?
        - What allowances are made for water wastage by pigs?

Step 2: Preparing the medication solution

- Do you prepare medication solutions using the same water source that pigs drink or do you use a higher quality water source e.g. rainwater?
- *If farm uses header tanks:*
    - Where exactly are the header tanks located? How easy are they to safely access?
    - Do you know the exact volume of water held in the tank servicing each weaner and grower/finisher building? (litres)
    - How many hours' pig water usage does each tank hold?
    - How exactly do you mix the medication product in water?
    - When and how exactly are top-up boluses of medication added? Please describe process
    - Are there any other challenges / risks / problems when preparing the medication solution?
- *If farm uses water or electric dosing pumps:*
    - What is the water flow rate at the doing pump?
    - What ratio of each medication product to water do you use for the stock solution and why?
    - What type(s) of container(s) do you use for the stock solution? What capacities? (litres)
    - Are there some particular medication products (or combinations of products) which are difficult to dissolve and keep in solution? Please describe
    - Do you use an agitator in the stock solution container? Why / why not?
    - Are there any other challenges / risks / problems when preparing the medication solution?

Step 3: Administering the medication solution to pigs

- At what time of day do you prefer to commence a water medication dosing event? Why?
- For each of the weaner and grower/finisher buildings you described in the questionnaire and other weaner and grower/finisher buildings on the farm, based on the configuration of the water supply system and dosing system, how long do you estimate it takes (minutes / hours) for the medicated solution to reach the closest pen of pigs and the pen furthest away? How confident are you of this?
- How long does each water medication dosing event continue for? (Hours / days). Why?
- Is the water line and pump (if used) sanitised after each water medication dosing event? What with?
- *If farm uses header tanks:*
    - Can you recall an incident in the past few months or years when the dosing system failed to deliver the correct dose to pigs due to an equipment failure or human error? What happened?
    - How often is each tank inspected and cleaned? How is this done?
    - Are there any other challenges / risks / problems when using headed tanks to medicate pigs?
- *If farm uses water or electric dosing pumps:*
    - How easy is the doser to adjust?
    - Is the doser fitted with an alarm to indicate if stock solution has run out?
    - Can you recall an incident in the past few months or years when the dosing system failed to deliver the correct dose to pigs due to an equipment failure or human error? What happened?
    - How often is the dosing system inspected and cleaned? (Pipes, pinch tubing etc.) How is this done?
    - How often is each doser serviced and calibrated?
    - If you have experience with both water or electric dosing pumps, what are the strengths and limitations of each type of pump?
    - Are there any other challenges / risks / problems when using dosing pumps to medicate pigs?
- How satisfied are you (score out of 10) that the majority of pigs in each of the weaner and grower/finisher buildings you described in the questionnaire consume the intended amount of antibiotic when dosed?
- What do you think are the most important factors influencing the degree of consistency in the dose of antibiotic consumed between pigs within a given weaner or grower/finisher shed?

4. Close of interview

Closing question

"We have covered a lot of ground and you have provided some really useful information and comments, so thank you very much! Is there anything else that you would like to add or think we haven't covered?"

Turn off the recorder

Closing statement

- Remind participant that they will receive a brief report summarising key points from the survey when all surveys have been completed and data have been analysed
- Thank interviewee and conclude interview

Post interview comments and/or observations
