# Supplementary material for: In-Water Antibiotic Dosing Practices on Pig Farms
Source: Antibiotics (Basel). 2021 Feb 8;10(2):169. doi: 10.3390/antibiotics10020169 (PMC7915319; doi:10.3390/antibiotics10020169)
Supplement: Supplementary file 1 [file antibiotics-10-00169-s001.zip › S3 - Final coding framework (NVIVO).pdf]

In-water antibiotic dosing practices on pig farms.
Stephen B. Little, Andrew P. Woodward, Glenn F. Browning and Helen Billman-Jacobe

Final coding framework (NVivo™)

Infrastructure
- Water sources
- On-farm water storages
- Pipelines and fittings
- Drinking appliances in pens
- Strategies used to improve [changes]

Water supply and usage by pigs
- Day's usage in on-farm storage
- Pigs' daily water demand
- Peak demand periods each day
- Water wastage by pigs
- Water quality
- Hygiene management
- Problems encountered with water system and dosing equipment

---

Antibiotic administration to pigs
- Dosing equipment
- Vet scripts and dosage calculations
- Timing and duration of dosing events
    - Outbreaks vs. metaphylaxis
- Antibiotics and other products administered
    - In-water vs. in-feed
- Problems / preferences
- Effectiveness of dosing events
    - Factors contributing to variability in antibiotic exposure among pigs in a group
- Strategies used to improve [changes]
