# Supplementary material for: In-Water Antibiotic Dosing Practices on Pig Farms
Source: Antibiotics (Basel). 2021 Feb 8;10(2):169. doi: 10.3390/antibiotics10020169 (PMC7915319; doi:10.3390/antibiotics10020169)
Supplement: Supplementary file 1 [file antibiotics-10-00169-s001.zip › S4 - In-water dosing practices and impacts.pdf]

In-water antibiotic dosing practices on pig farms.

Stephen B. Little, Andrew P. Woodward, Glenn F. Browning and Helen Billman-Jacobe

Summary table:
Specific in-water antibiotic dosing practices, their consequences and the expected impact on pigs' systemic exposure to an antibiotic administered through their drinking water

**In-water dosing practice**	**Consequences and expected impact on pigs' systemic exposure to antibiotic**
Header tanks are used to dose pigs with each tank's inlet valve deliberately left open so tank continuously refills with unmedicated water	Concentration of antibiotic in water flowing from tank along water line to pigs is progressively reduced over time. Pharmacokinetic /pharmacodynamic (PK/PD) index attained by pigs may not be high. Many / all pigs are under-dosed
Very highly concentrated dosing pump stock solution is prepared of an antibiotic product with low solubility such as amoxicillin tryhydrate, trimethoprim-sulphadiazine, chlortetracycline hydrochloride, for use with a short dosing period and/or low injection ratio	Many undissolved antibiotic particles are present which are drawn up by the pump and injected into water line, especially early in the dosing event. Many / all pigs do not ingest sufficient antibiotic, and antibiotic absorption and distribution in pigs is also compromised i.e. are under-dosed
Dosing pump is located in central position on farm, requiring medicated water to travel long distances to pig building	Antibiotic is prone to degradation if exposed to external factors that adversely affect its stability e.g. water quality parameters, water pipe materials. If so, many / all pigs do not ingest sufficient active antibiotic i.e. are under-dosed
Preventative maintenance is not done on dosing pump. Pump performs poorly or fails during dosing event and this is not detected and rectified by farm staff for some hours	Concentration of antibiotic in water line fluctuates over time or falls abruptly to nil. Many / all pigs do not ingest sufficient antibiotic i.e. are under-dosed
An assumed water wastage value is used in dosing calculations which is an under-estimate of pigs' actual water wastage rate	Many / all pigs do not ingest sufficient antibiotic i.e. are under-dosed
An assumed water wastage value is used in dosing calculations which is an over-estimate of pigs' actual water wastage rate	Many / all pigs ingest excessive antibiotic i.e. are over-dosed
Dosing event commencement time is chosen without using measured data on pigs' daily water usage pattern. Dosing event is commenced during a period of low water consumption by pigs	Ingestion of antibiotic in first few hours is low. Plasma concentration of antibiotic in pigs therefore rises slowly and the PK/PD index attained by pigs may not be high. Many / all pigs are under-dosed

For further information on sources of between-animal variability in systemic exposure to an antibiotic when water medicating pigs, please refer to:

Little, S.B., Crabb, H.K., Woodward, A.P., Browning, G.F. and Billman-Jacobe, H. Review: Water medication of growing pigs: sources of between-animal variability in systemic exposure to antimicrobials. Animal 2019, 13(12), 3031-3040. http://doi.org/10.1017/S1751731119001903
